# 1-(4-Methyl­benzo­yl)-3-[5-(4-pyrid­yl)-1,3,4-thia­diazol-2-yl]urea

**DOI:** 10.1107/S1600536808035149

**Published:** 2008-11-08

**Authors:** Xiu-Huan Zhan, Zi-Yun Wang, Xiao-Hong Tan, Zhi-Wei Tan, Xin-Jian Song

**Affiliations:** a Department of Chemistry, Zhoukou Normal University, Zhoukou 466000, People’s Republic of China; b Key Laboratory of Biological Resources Protection and Utilization of Hubei Province, Hubei University for Nationalities, Enshi, Hubei 445000, People’s Republic of China; c School of Chemical and Environmental Engineering, Hubei University for Nationalities, Enshi, Hubei 445000, People’s Republic of China

## Abstract

In the title compound, C_16_H_13_N_5_O_2_S, the five non-H atoms of the urea linkage adopt a planar configuration owing to the presence of an intra­molecular N—H⋯O hydrogen bond. The maximum deviation from planarity is 0.022 (2) Å. The thia­diazole and pyridine heterocyclic rings are close to being coplanar, with a dihedral angle of 6.7 (2)° between their mean planes. Inter­molecular N—H⋯O hydrogen bonds link two neighbouring mol­ecules into centrosymmetric *R*_2_^2^(8) dimers. Four C atoms and the attached H atoms of the benzene ring are disordered over two positions of equal occupancy.

## Related literature

For general background, see: Chen *et al.* (2005 ▶); Foroumadi *et al.* (2002 ▶); Song *et al.* (2007 ▶); Song *et al.* (2008 ▶). For related structures, see: Song & Tan *et al.* (2005 ▶). For the synthesis, see: Song & Feng *et al.* (2005 ▶).
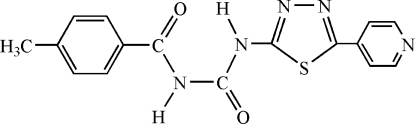


## Experimental

### 

#### Crystal data

C_16_H_13_N_5_O_2_S
*M* *_r_* = 339.37
Triclinic, 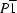

*a* = 5.0563 (5) Å
*b* = 11.8561 (11) Å
*c* = 13.2506 (12) Å
α = 88.892 (2)°
β = 80.849 (2)°
γ = 77.989 (2)°
*V* = 766.99 (13) Å^3^
*Z* = 2
Mo *K*α radiation
μ = 0.23 mm^−1^
*T* = 297 (2) K
0.20 × 0.10 × 0.04 mm

#### Data collection

Bruker SMART CCD area-detector diffractometer
Absorption correction: none
6171 measured reflections
2978 independent reflections
2120 reflections with *I* > 2σ(*I*)
*R*_int_ = 0.096

#### Refinement

*R*[*F* ^2^ > 2σ(*F* ^2^)] = 0.062
*wR*(*F* ^2^) = 0.195
*S* = 1.06
2978 reflections
214 parameters
H-atom parameters constrained
Δρ_max_ = 0.24 e Å^−3^
Δρ_min_ = −0.32 e Å^−3^

### 

Data collection: *SMART* (Bruker, 2001 ▶); cell refinement: *SAINT* (Bruker, 2001 ▶); data reduction: *SAINT*; program(s) used to solve structure: *SHELXS97* (Sheldrick, 2008 ▶); program(s) used to refine structure: *SHELXL97* (Sheldrick, 2008 ▶); molecular graphics: *SHELXTL* (Sheldrick, 2008 ▶); software used to prepare material for publication: *SHELXTL*.

## Supplementary Material

Crystal structure: contains datablocks global, I. DOI: 10.1107/S1600536808035149/fj2164sup1.cif

Structure factors: contains datablocks I. DOI: 10.1107/S1600536808035149/fj2164Isup2.hkl

Additional supplementary materials:  crystallographic information; 3D view; checkCIF report

## Figures and Tables

**Table 1:** Hydrogen-bond geometry (Å, °)

*D*—H⋯*A*	*D*—H	H⋯*A*	*D*⋯*A*	*D*—H⋯*A*
N2—H2⋯O1	0.86	1.90	2.583 (3)	135
N1—H1⋯O2^i^	0.86	2.10	2.935 (3)	165

Symmetry code: (i) 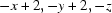.

**Fig. 1.**
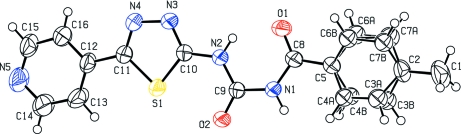
View of the molecule of (I) showing the atom-labelling scheme. Displacement ellipsoids are drawn at the 50% probability level. H atoms are represented by circles of arbitrary size. Both disorder components are shown.

**Fig. 2.**
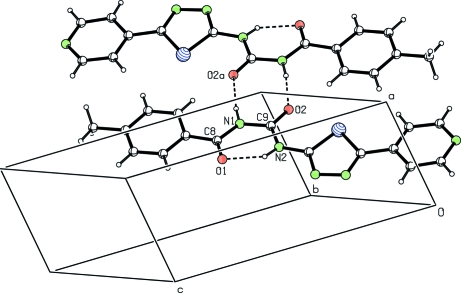
A partial packing diagram for (I) [symmetry code: (a) 2 - x, 2 - y, -z]. Hydrogen bonds are indicated by dashed lines.

## supplementary crystallographic information

### Comment

1,3,4-Thiadiazole derivatives have been reported to possess broad spectrum bioactivities (Foroumadi *et al.*, 2002; Song *et al.*, 2007). Urea derivatives, especially aroylurea derivatives, have attracted many chemist's interest owing to their diverse biological effects, such as insecticidal, fungicidal, herbicidal and plant-growth regulating activities (Chen *et al.*, 2005; Song *et al.*, 2008). Furthermore, pyridine derivatives have become one of research hotspots in modern agrochemistry and medicinal chemistry. In our continuing search for new plant-growth regulators, we would like to investigate aroyl ureas incorporating both 1,3,4-thiadiazole and pyridine rings, including the title compound.

The crystal structure (Fig.1) revealed that the urea linkage unit O1—C8—N1—C9—N2—H2 adopts the stable conformation due to the the formation of a strong intramolecular N—H···O hydrogen bond to give a planar six-membered ring, as reported (Song and Tan *et al.*, 2005), which is essentially coplanar with a maximum deviation from planarity of 0.022 (2)Å for atom O1. The thiadiazole and pyridine heterocyclic fragments also lie essentially in the same plane, the maximum deviation from that plane being 0.059 (3)Å for atom C15. The dihedral angle between the two mean planes is 6.7 (2)°. All bond lengths and angles are as expected. In the crystal structure, the molecules are linked by complementary N—H···O hydrogen bonds into centrosymmetric *R*_2_^2^(8) dimers (Fig. 2 and Table 1).

### Experimental

The title compound  was prepared according to the procedure of Song and Feng *et al.* (2005). Suitable crystals were obtained by vapor diffusion of methanol in DMF at room temperature (m.p. >573 K). Elemental analysis: analysis calculated for C_16_H_13_N_5_O_2_S: C 56.63, H 3.86, N 20.64%; found: C 56.55, H 3.76, N 20.81%.

### Refinement

All H atoms were initially located in a difference Fourier map. The methyl H atoms were then constrained to an ideal geometry with C—H distances of 0.96 Å and *U*_iso_(H) = 1.5*U*_eq_(C), but each group was allowed to rotate freely about its C—C bond. Other H atoms were positioned geometrically and constrained to ride on their parent atoms with C—H distances of 0.93 Å, N—H distances of 0.86Å and *U*_iso_(H) = 1.2*U*_eq_(C). The atoms C3, C4, C6 and C7 in the benzene ring are found to be disordered over two positions.

### Crystal data

**Table:**

C_16_H_13_N_5_O_2_S_1_	*Z* = 2
*M**_r_* = 339.37	*F*_000_ = 352
Triclinic, *P*1	*D*_x_ = 1.469 Mg m^−^^3^
Hall symbol: -P 1	Melting point > 573 K
*a* = 5.0563 (5) Å	Mo *K*α radiation λ = 0.71073 Å
*b* = 11.8561 (11) Å	Cell parameters from 1721 reflections
*c* = 13.2506 (12) Å	θ = 2.3–24.7º
α = 88.892 (2)º	µ = 0.23 mm^−^^1^
β = 80.849 (2)º	*T* = 297 (2) K
γ = 77.989 (2)º	Block, colorless
*V* = 766.99 (13) Å^3^	0.20 × 0.10 × 0.04 mm

### Data collection

**Table:**

Bruker SMART CCD area-detector diffractometer	2120 reflections with *I* > 2σ(*I*)
Radiation source: fine-focus sealed tube	*R*_int_ = 0.096
Monochromator: graphite	θ_max_ = 26.0º
*T* = 297(2) K	θ_min_ = 1.8º
φ and ω scans	*h* = −6→6
Absorption correction: none	*k* = −14→14
6171 measured reflections	*l* = −16→16
2978 independent reflections	

### Refinement

**Table:**

Refinement on *F*^2^	Secondary atom site location: difference Fourier map
Least-squares matrix: full	Hydrogen site location: inferred from neighbouring sites
*R*[*F*^2^ > 2σ(*F*^2^)] = 0.062	H-atom parameters constrained
*wR*(*F*^2^) = 0.195	*w* = 1/[σ^2^(*F*_o_^2^) + (0.1026*P*)^2^] where *P* = (*F*_o_^2^ + 2*F*_c_^2^)/3
*S* = 1.06	(Δ/σ)_max_ < 0.001
2978 reflections	Δρ_max_ = 0.24 e Å^−^^3^
214 parameters	Δρ_min_ = −0.32 e Å^−^^3^
Primary atom site location: structure-invariant direct methods	Extinction correction: none

### Special details

**Table:**

Geometry. All e.s.d.'s (except the e.s.d. in the dihedral angle between two l.s. planes) are estimated using the full covariance matrix. The cell e.s.d.'s are taken into account individually in the estimation of e.s.d.'s in distances, angles and torsion angles; correlations between e.s.d.'s in cell parameters are only used when they are defined by crystal symmetry. An approximate (isotropic) treatment of cell e.s.d.'s is used for estimating e.s.d.'s involving l.s. planes.
Refinement. Refinement of *F*^2^ against ALL reflections. The weighted *R*-factor *wR* and goodness of fit *S* are based on *F*^2^, conventional *R*-factors *R* are based on *F*, with *F* set to zero for negative *F*^2^. The threshold expression of *F*^2^ > σ(*F*^2^) is used only for calculating *R*-factors(gt) *etc*. and is not relevant to the choice of reflections for refinement. *R*-factors based on *F*^2^ are statistically about twice as large as those based on *F*, and *R*- factors based on ALL data will be even larger.

### Fractional atomic coordinates and isotropic or equivalent isotropic displacement parameters (Å^2^)

**Table:**

	*x*	*y*	*z*	*U*_iso_*/*U*_eq_	Occ. (<1)
S1	0.59211 (17)	0.73166 (7)	−0.09171 (5)	0.0562 (3)	
N1	0.8010 (5)	0.96100 (19)	0.13767 (16)	0.0449 (6)	
H1	0.9109	1.0058	0.1159	0.054*	
N2	0.5583 (5)	0.82578 (19)	0.09796 (16)	0.0459 (6)	
H2	0.4995	0.8259	0.1625	0.055*	
N3	0.2887 (6)	0.6962 (2)	0.07332 (18)	0.0565 (7)	
N4	0.2335 (5)	0.6273 (2)	0.00007 (19)	0.0537 (7)	
N5	0.3212 (7)	0.4482 (3)	−0.3539 (2)	0.0800 (9)	
O1	0.5680 (6)	0.8977 (2)	0.27987 (16)	0.0916 (9)	
O2	0.8225 (5)	0.90478 (17)	−0.02610 (13)	0.0574 (6)	
C1	1.0069 (10)	1.2823 (3)	0.5070 (3)	0.0936 (14)	
H1A	0.9255	1.2708	0.5759	0.140*	
H1B	0.9400	1.3601	0.4874	0.140*	
H1C	1.2024	1.2684	0.5025	0.140*	
C2	0.9326 (7)	1.1995 (3)	0.4361 (2)	0.0567 (8)	
C5	0.8036 (6)	1.0456 (2)	0.3046 (2)	0.0460 (7)	
C3A	0.9463 (13)	1.2164 (5)	0.3343 (4)	0.0512 (14)*	0.50
H3A	0.9870	1.2852	0.3082	0.061*	0.50
C4A	0.9036 (12)	1.1382 (5)	0.2677 (4)	0.0432 (13)*	0.50
H4A	0.9429	1.1484	0.1976	0.052*	0.50
C6A	0.7810 (12)	1.0218 (5)	0.4113 (4)	0.0501 (14)*	0.50
H6A	0.7357	0.9534	0.4365	0.060*	0.50
C7A	0.8276 (13)	1.1023 (6)	0.4758 (5)	0.0533 (15)*	0.50
H7A	0.7903	1.0933	0.5462	0.064*	0.50
C3B	1.1141 (12)	1.1626 (5)	0.3379 (4)	0.0473 (13)*	0.50
H3B	1.2799	1.1857	0.3197	0.057*	0.50
C4B	1.0226 (12)	1.0914 (5)	0.2743 (4)	0.0405 (12)*	0.50
H4B	1.1179	1.0753	0.2084	0.049*	0.50
C6B	0.6374 (12)	1.0858 (5)	0.3994 (4)	0.0464 (13)*	0.50
H6B	0.4711	1.0634	0.4188	0.056*	0.50
C7B	0.7220 (13)	1.1576 (5)	0.4624 (4)	0.0468 (14)*	0.50
H7B	0.6200	1.1760	0.5270	0.056*	0.50
C8	0.7154 (7)	0.9624 (3)	0.2418 (2)	0.0518 (8)	
C9	0.7317 (6)	0.8962 (2)	0.0638 (2)	0.0430 (7)	
C10	0.4699 (6)	0.7538 (2)	0.03602 (19)	0.0422 (6)	
C11	0.3759 (6)	0.6354 (2)	−0.0884 (2)	0.0454 (7)	
C12	0.3576 (6)	0.5700 (2)	−0.1797 (2)	0.0503 (7)	
C13	0.5012 (9)	0.5865 (4)	−0.2729 (3)	0.1010 (16)	
H13	0.6168	0.6387	−0.2796	0.121*	
C14	0.4754 (10)	0.5258 (4)	−0.3576 (3)	0.1101 (18)	
H14	0.5720	0.5405	−0.4206	0.132*	
C15	0.1854 (7)	0.4317 (3)	−0.2624 (3)	0.0663 (9)	
H15	0.0759	0.3771	−0.2572	0.080*	
C16	0.1956 (7)	0.4896 (3)	−0.1754 (2)	0.0581 (8)	
H16	0.0937	0.4747	−0.1136	0.070*	

### Atomic displacement parameters (Å^2^)

**Table:**

	*U*^11^	*U*^22^	*U*^33^	*U*^12^	*U*^13^	*U*^23^
S1	0.0657 (6)	0.0700 (6)	0.0411 (5)	−0.0402 (4)	0.0034 (3)	−0.0127 (3)
N1	0.0545 (15)	0.0513 (13)	0.0352 (12)	−0.0283 (11)	−0.0024 (10)	−0.0022 (9)
N2	0.0566 (15)	0.0543 (14)	0.0333 (12)	−0.0283 (12)	−0.0042 (10)	−0.0003 (10)
N3	0.0751 (18)	0.0661 (16)	0.0403 (13)	−0.0439 (14)	−0.0060 (12)	0.0019 (11)
N4	0.0653 (17)	0.0581 (15)	0.0480 (14)	−0.0341 (13)	−0.0116 (12)	0.0018 (11)
N5	0.093 (2)	0.082 (2)	0.073 (2)	−0.0397 (18)	−0.0040 (17)	−0.0306 (16)
O1	0.145 (2)	0.119 (2)	0.0372 (12)	−0.0999 (19)	0.0036 (13)	−0.0042 (12)
O2	0.0811 (15)	0.0682 (13)	0.0326 (10)	−0.0449 (12)	0.0014 (9)	−0.0052 (9)
C1	0.156 (4)	0.078 (3)	0.062 (2)	−0.043 (3)	−0.035 (2)	−0.0158 (19)
C2	0.074 (2)	0.0541 (18)	0.0452 (17)	−0.0130 (16)	−0.0175 (15)	−0.0104 (13)
C5	0.0518 (17)	0.0528 (16)	0.0355 (14)	−0.0176 (13)	−0.0036 (12)	−0.0009 (12)
C8	0.066 (2)	0.0588 (18)	0.0353 (15)	−0.0287 (15)	−0.0031 (13)	−0.0002 (12)
C9	0.0544 (17)	0.0432 (15)	0.0353 (14)	−0.0201 (13)	−0.0055 (12)	−0.0010 (11)
C10	0.0485 (16)	0.0447 (15)	0.0374 (14)	−0.0173 (12)	−0.0088 (12)	0.0017 (11)
C11	0.0501 (17)	0.0461 (15)	0.0440 (16)	−0.0178 (13)	−0.0082 (13)	−0.0026 (12)
C12	0.0527 (18)	0.0505 (16)	0.0534 (18)	−0.0203 (14)	−0.0114 (14)	−0.0095 (13)
C13	0.125 (4)	0.136 (4)	0.063 (2)	−0.099 (3)	0.022 (2)	−0.037 (2)
C14	0.137 (4)	0.154 (4)	0.059 (2)	−0.099 (4)	0.023 (2)	−0.043 (2)
C15	0.079 (2)	0.0558 (19)	0.075 (2)	−0.0306 (18)	−0.0219 (19)	−0.0083 (16)
C16	0.073 (2)	0.0506 (17)	0.0576 (19)	−0.0263 (16)	−0.0127 (16)	−0.0013 (13)

### Geometric parameters (Å, °)

**Table:**

S1—C10	1.712 (3)	C5—C6B	1.425 (6)
S1—C11	1.733 (3)	C5—C6A	1.427 (6)
N1—C8	1.378 (3)	C5—C8	1.483 (4)
N1—C9	1.386 (3)	C3A—C4A	1.363 (8)
N1—H1	0.8600	C3A—H3A	0.9300
N2—C9	1.354 (3)	C4A—H4A	0.9300
N2—C10	1.380 (3)	C6A—C7A	1.374 (8)
N2—H2	0.8600	C6A—H6A	0.9300
N3—C10	1.285 (3)	C7A—H7A	0.9300
N3—N4	1.379 (3)	C3B—C4B	1.397 (8)
N4—C11	1.287 (3)	C3B—H3B	0.9300
N5—C14	1.320 (5)	C4B—H4B	0.9300
N5—C15	1.327 (4)	C6B—C7B	1.378 (8)
O1—C8	1.225 (4)	C6B—H6B	0.9300
O2—C9	1.217 (3)	C7B—H7B	0.9300
C1—C2	1.512 (4)	C11—C12	1.475 (4)
C1—H1A	0.9600	C12—C13	1.362 (4)
C1—H1B	0.9600	C12—C16	1.375 (4)
C1—H1C	0.9600	C13—C14	1.382 (5)
C2—C7B	1.264 (6)	C13—H13	0.9300
C2—C3A	1.353 (6)	C14—H14	0.9300
C2—C7A	1.424 (7)	C15—C16	1.367 (5)
C2—C3B	1.483 (6)	C15—H15	0.9300
C5—C4B	1.335 (6)	C16—H16	0.9300
C5—C4A	1.353 (6)		
			
C10—S1—C11	86.09 (12)	C6A—C7A—H7A	119.7
C8—N1—C9	127.9 (2)	C2—C7A—H7A	119.7
C8—N1—H1	116.1	C4B—C3B—C2	116.5 (5)
C9—N1—H1	116.1	C4B—C3B—H3B	121.8
C9—N2—C10	124.4 (2)	C2—C3B—H3B	121.8
C9—N2—H2	117.8	C5—C4B—C3B	122.4 (5)
C10—N2—H2	117.8	C5—C4B—H4B	118.8
C10—N3—N4	111.7 (2)	C3B—C4B—H4B	118.8
C11—N4—N3	112.8 (2)	C7B—C6B—C5	120.3 (5)
C14—N5—C15	115.8 (3)	C7B—C6B—H6B	119.9
C2—C1—H1A	109.5	C5—C6B—H6B	119.9
C2—C1—H1B	109.5	C2—C7B—C6B	122.7 (5)
H1A—C1—H1B	109.5	C2—C7B—H7B	118.7
C2—C1—H1C	109.5	C6B—C7B—H7B	118.7
H1A—C1—H1C	109.5	O1—C8—N1	120.4 (3)
H1B—C1—H1C	109.5	O1—C8—C5	121.8 (3)
C7B—C2—C3A	105.2 (4)	N1—C8—C5	117.7 (2)
C3A—C2—C7A	116.6 (4)	O2—C9—N2	123.3 (3)
C7B—C2—C3B	119.8 (4)	O2—C9—N1	120.5 (2)
C7A—C2—C3B	107.3 (4)	N2—C9—N1	116.2 (2)
C7B—C2—C1	120.0 (4)	N3—C10—N2	120.5 (2)
C3A—C2—C1	122.8 (4)	N3—C10—S1	115.5 (2)
C7A—C2—C1	120.5 (4)	N2—C10—S1	124.0 (2)
C3B—C2—C1	120.1 (4)	N4—C11—C12	123.7 (3)
C4B—C5—C6B	117.5 (4)	N4—C11—S1	114.0 (2)
C4A—C5—C6B	104.1 (4)	C12—C11—S1	122.3 (2)
C4B—C5—C6A	110.0 (4)	C13—C12—C16	116.6 (3)
C4A—C5—C6A	119.8 (4)	C13—C12—C11	121.4 (3)
C4B—C5—C8	123.5 (3)	C16—C12—C11	122.0 (3)
C4A—C5—C8	124.9 (3)	C12—C13—C14	120.2 (3)
C6B—C5—C8	118.9 (3)	C12—C13—H13	119.9
C6A—C5—C8	115.3 (3)	C14—C13—H13	119.9
C2—C3A—C4A	123.7 (5)	N5—C14—C13	123.5 (4)
C2—C3A—H3A	118.1	N5—C14—H14	118.2
C4A—C3A—H3A	118.1	C13—C14—H14	118.2
C5—C4A—C3A	119.4 (5)	N5—C15—C16	124.3 (3)
C5—C4A—H4A	120.3	N5—C15—H15	117.8
C3A—C4A—H4A	120.3	C16—C15—H15	117.8
C7A—C6A—C5	118.5 (5)	C15—C16—C12	119.6 (3)
C7A—C6A—H6A	120.8	C15—C16—H16	120.2
C5—C6A—H6A	120.8	C12—C16—H16	120.2
C6A—C7A—C2	120.7 (5)		
			
C10—N3—N4—C11	0.4 (4)	C9—N1—C8—O1	3.1 (5)
C7B—C2—C3A—C4A	−43.7 (7)	C9—N1—C8—C5	−175.8 (3)
C7A—C2—C3A—C4A	−9.7 (8)	C4B—C5—C8—O1	158.9 (4)
C3B—C2—C3A—C4A	75.1 (7)	C4A—C5—C8—O1	−162.1 (4)
C1—C2—C3A—C4A	174.0 (5)	C6B—C5—C8—O1	−25.6 (5)
C4B—C5—C4A—C3A	−89.2 (8)	C6A—C5—C8—O1	18.8 (5)
C6B—C5—C4A—C3A	30.4 (7)	C4B—C5—C8—N1	−22.2 (5)
C6A—C5—C4A—C3A	−9.0 (8)	C4A—C5—C8—N1	16.8 (6)
C8—C5—C4A—C3A	172.0 (4)	C6B—C5—C8—N1	153.3 (3)
C2—C3A—C4A—C5	10.0 (9)	C6A—C5—C8—N1	−162.3 (3)
C4B—C5—C6A—C7A	42.4 (7)	C10—N2—C9—O2	1.7 (5)
C4A—C5—C6A—C7A	8.5 (7)	C10—N2—C9—N1	−179.0 (2)
C6B—C5—C6A—C7A	−66.8 (6)	C8—N1—C9—O2	179.4 (3)
C8—C5—C6A—C7A	−172.3 (5)	C8—N1—C9—N2	0.1 (5)
C5—C6A—C7A—C2	−8.6 (9)	N4—N3—C10—N2	−178.3 (2)
C7B—C2—C7A—C6A	86.0 (9)	N4—N3—C10—S1	0.4 (3)
C3A—C2—C7A—C6A	9.0 (8)	C9—N2—C10—N3	−174.6 (3)
C3B—C2—C7A—C6A	−32.1 (7)	C9—N2—C10—S1	6.8 (4)
C1—C2—C7A—C6A	−174.6 (5)	C11—S1—C10—N3	−0.7 (2)
C7B—C2—C3B—C4B	6.7 (7)	C11—S1—C10—N2	177.9 (3)
C3A—C2—C3B—C4B	−70.3 (7)	N3—N4—C11—C12	179.5 (3)
C7A—C2—C3B—C4B	40.9 (6)	N3—N4—C11—S1	−1.0 (3)
C1—C2—C3B—C4B	−176.4 (4)	C10—S1—C11—N4	1.0 (2)
C4A—C5—C4B—C3B	81.4 (8)	C10—S1—C11—C12	−179.5 (3)
C6B—C5—C4B—C3B	9.6 (8)	N4—C11—C12—C13	176.2 (3)
C6A—C5—C4B—C3B	−33.0 (7)	S1—C11—C12—C13	−3.3 (5)
C8—C5—C4B—C3B	−174.9 (4)	N4—C11—C12—C16	−3.7 (5)
C2—C3B—C4B—C5	−8.8 (8)	S1—C11—C12—C16	176.7 (2)
C4B—C5—C6B—C7B	−8.2 (7)	C16—C12—C13—C14	1.4 (7)
C4A—C5—C6B—C7B	−39.6 (6)	C11—C12—C13—C14	−178.6 (4)
C6A—C5—C6B—C7B	80.4 (7)	C15—N5—C14—C13	0.8 (8)
C8—C5—C6B—C7B	176.0 (5)	C12—C13—C14—N5	−1.8 (9)
C3A—C2—C7B—C6B	33.5 (7)	C14—N5—C15—C16	0.5 (6)
C7A—C2—C7B—C6B	−82.0 (8)	N5—C15—C16—C12	−0.8 (6)
C3B—C2—C7B—C6B	−6.0 (8)	C13—C12—C16—C15	−0.2 (5)
C1—C2—C7B—C6B	177.1 (5)	C11—C12—C16—C15	179.7 (3)
C5—C6B—C7B—C2	6.8 (9)		

### Hydrogen-bond geometry (Å, °)

**Table:**

*D*—H···*A*	*D*—H	H···*A*	*D*···*A*	*D*—H···*A*
N2—H2···O1	0.86	1.90	2.583 (3)	135
N1—H1···O2^i^	0.86	2.10	2.935 (3)	165

Symmetry codes: (i) −*x*+2, −*y*+2, −*z*.
